# Improving the informatics competency of critical care nurses: results of an interventional study in the southeast of Iran

**DOI:** 10.1186/s12911-020-01244-5

**Published:** 2020-09-11

**Authors:** Somayeh Jouparinejad, Golnaz Foroughameri, Reza Khajouei, Jamileh Farokhzadian

**Affiliations:** 1 Student Research Committee, Kerman University of Medical Sciences, Kerman, Iran; 2 Nursing Research Center, Kerman University of Medical Sciences, PO Box: 7716913555, Kerman, Iran; 3 Department of Community Health Nursing, School of Nursing and Midwifery, Kerman University of Medical Sciences, Kerman, Iran; 4 Medical Informatics Research Center, Institute for Futures Studies in Health, Kerman University of Medical Sciences, Kerman, Iran; 5 Department of Health Information Sciences, Faculty of Management and Medical Information Sciences, Kerman University of Medical Sciences, Kerman, Iran

**Keywords:** Competence, Education, Health information technology, Nursing informatics, Critical care settings

## Abstract

**Background:**

Nursing informatics (NI) along with growth and development of health information technology (HIT) is becoming a fundamental part of all domains of nursing practice especially in critical care settings. Nurses are expected to equip with NI competency for providing patient-centered evidence-based care. Therefore, it is important and necessary to improve nurses’ NI competency through educational programs for effective using of HIT. This study aimed to evaluate the impact of a training program on NI competency of critical care nurses.

**Methods:**

This interventional study was conducted in 2019. Stratified sampling technique was used to select 60 nurses working in critical care units of three hospitals affiliated with a large University of Medical Sciences in the southeast of Iran. These nurses were assigned randomly and equally to the control and intervention groups. NI competency was trained to the intervention group in a three-day workshop. Data were collected using demographic questionnaire and the adapted Nursing Informatics Competency Assessment Tool (NICAT) before and 1 month after the intervention. Rahman in the US (2015) developed and validated the original NICAT to assess self-reported NI competency of nurses with 30 items and three dimensions (Computer literacy, Informatics literacy Information management skills). The NICAT is scored on a five-point Likert scale and the overall score ranges from 30 to150. Two medical informatics specialists and eight nursing faculty members approved the validity of the adapted version of NICAT and its reliability was confirmed by Cronbach’s alpha (95%).

**Results:**

All 60 participants completed the educational program and returned the completed questionnaire**.** Majority of participants in the intervention and control groups were female (83.30%), married nurses (70.90, 73.30%) aged 30–40 years (51.6, 35.5%). In the pretest stage, both intervention and control groups were competent in terms of the NI competency and its dimensions, and no significant difference was observed between them (*p* = 0.65). However, in the posttest, the NI competency and its dimensions in the intervention group significantly increased with a large effect size compared with the control group (*p* = 0.001). This difference showed that the intervention group was proficient in the posttest stage. The highest mean difference in the intervention group was associated with the informatics literacy dimension and the lowest mean difference was associated with the informatics management skills dimension.

**Conclusions:**

The improved scores of NI competency and its dimensions after using the training program implied the effectiveness of this method in enhancing the NI competency of nurses working in the critical care units. The application of the training program in diverse domains of nursing practice shows its high efficiency. The project is fundamental for improving nurses’ NI competency through continuous educational programs in Iran, other cultures and contexts.

## Background

Since the beginning of the third millennium, health information technology (HIT) has dramatically influenced all aspects of healthcare [1]. HIT plays an important role in nursing practice, and nurses have participated in the conception, design and implementation of HIT. Following the development of information technology initiatives in nursing, the nursing informatics (NI) discipline has been emerged due to the expansion of the knowledge required in this field [2]. NI is a specialty that integrates nursing science, computer science, and information science to manage and communicate data, information, and knowledge in nursing practice [3, 4].

Several studies suggested that NI integrates the best practices into patient care [5] and supports evidence-based practice (EBP) [6]. Hence, it has positive effects on healthcare and nursing practice such as enhancing patient safety, improving healthcare quality, and reducing healthcare costs. NI facilitates exchange of information between patients and nurses, and improves their relationship [7]. It helps consumers, patients, nurses, and other healthcare team to make appropriate decision and to achieve favorable outcomes [4].

Despite the well-documented positive impacts of HIT, several studies reported that the introduction of HIT into clinical settings is associated with unintended and unanticipated negative impacts, such as adverse patient outcomes, patient safety risks, and patient harm [8–10]. Other negative consequences include clinicians burnout, HIT-related stress, dissatisfaction with HIT, inclination to reduce clinical working hours and quitting job or leaving current practice [11].

Researchers emphasized that low NI competency of nurses were associated with negative impacts of HIT in patient care [12]. NI competency is defined as the nurses’ knowledge, skills, and attitudes to collect, store, retrieve, process and use information in nursing care [13]. The NI competency ranges from simple clinical skills to complex application-based knowledge [14]. According to American Nurses Association (ANA), various nursing informatics activities are prescribed within four levels of nursing practice, including beginner nurse, experienced nurse, informatics nurse specialist, and informatics innovator [15]. The Technology Informatics Guiding Education Reform (TIGER) Initiative in 2009 put forward recommendations for NI competency of the bedside nurses and integration of the NI into nursing education and development of all nurses in every role such as beside nurse, nurse leader and nurse educators. The recommendations are in three categories, including computer literacy, information literacy, and informatics management skills [5, 13]. HIT would be ineffective in the healthcare settings without the existence of NI competency, imposing aforementioned negative impacts [14]. NI was considered as a requirement for EBP. When nurses have high levels of NI competency, they will have better readiness for EBP [16].

Nurses working in critical care units need NI competency much higher than other nursing groups because of the criticality of working in critical care settings. Patients in critical settings need sophisticated high-tech equipment, and specialized clinicians continuously monitor them. The patient safety and patient outcomes are more vital in such settings than in other units [13, 17–19]. The literature review has recognized the gap in NI competency required for nurses in different work environments and critical care settings. The results suggested that more training courses are necessary to increase NI competency and to better meet the competency requirements of nursing profession [17, 20–23]. Poor NI competency of both newly graduated and experienced nurses not only puts patients at risk for suboptimal care, but also places additional stresses on nurses who are expected to demonstrate improved patient outcomes by using HIT [12]. Therefore, nurses’ NI competency in the delivery of patient care should be improved with continuous learning. Without educational interventions, nurses are not able to effectively use HIT in practice [23–26]. In addition, newly graduated nurses in clinical settings reported that the NI education they received was insufficient, their instructors demonstrated incomplete understanding of NI, and the skills taught in these classes were not transferred well to the workplace [12]. Researchers have reported that nursing schools lack standardization to teach NI competency required for nursing students. The integration of NI competency into nursing education will require nursing curricula reform and development of innovative educational programs [25].

Finally, HIT is now available, and strategies such as research, educational interventions, and modules should be added to professional development of nurses. These strategies can support nurses to adapt with HIT in practice and reduce negative impacts of HIT. This process can be accomplished through developing and evaluating effective educational programs in different settings [7, 11]. Few studies implemented a training program to improve NI competency among nurses and reported that nurses’ NI competency increased after the educational intervention [4, 27, 28]. However, the improvement of the NI competency of critical care nurses in not sufficiently addressed in the literature. Furthermore, it seems that the NI competency depends on organizational context such as hospital status, size, and resource. The objective of this study was to evaluate the impact of a training program on NI competency of nurses working in critical care units in Iran.

## Materials and methods

### Study design and settings

The present study was an educational intervention to improve NI competency of nurses in critical care units from March to April 2019. The study was conducted using a pretest-posttest design with the intervention and control groups. The study settings were 18 critical care units, including10 intensive care units (ICUs), 6 coronary care units (CCUs), and 2 dialysis wards selected from three educational hospitals affiliated with Kerman University of Medical Sciences. Kerman University of Medical Sciences is the largest medical university in southeast Iran that has three large general hospitals. These hospitals have more than 1100 beds and admit patients from southeast of Iran, within the radius of 500 km. In the organizational chart of the Iranian hospitals, ICUs, CCUs, and dialysis wards are classified into critical care units [29], and nurses working in these units have the same benefits. According to the nursing curricula, nursing students pass critical courses in these settings. In addition, the distribution of nurses is similar in all Iranian hospitals. The majority of nurses have bachelor’s degrees and are recruited in all hospitals with the same pattern. There are no registered nurse (RN) and informatics nurse specialist in the study settings. According to the nurse manager, nurses working in critical settings are not fixed and have rotational shifts in many wards.

Experts in nursing education in Iran offered integration of NI competency and research in nursing into undergraduate nursing curricula in 2010. However, the undergraduate nursing curriculum included only 1.5 credits of research in nursing (34 h, in the third semester) and one credit of information technology in nursing (26 h, in the first semester). Although basic skills research and the use of computers may be taught in courses, the demands of the nursing curriculum do not allow for more education. Consequently, there are gaps in integration of the NI competency into nursing education. Furthermore, nurses graduated before 2010 have not received formal training on NI competency.

It is noteworthy that authorities of Kerman University of Medical Sciences select appropriate e-Health applications for the hospitals. For example, Hospital Information System (HIS) is currently implemented in nursing stations, pharmacy, laboratories, radiology, outpatient and inpatient wards, etc. Hospitals have implemented HIS to automate hospital management tasks such as documentation of nursing care, cost management, resource management, medication management, accounting, nurses and physician performance assessment, and patient registration, etc. Following the implementation of HIS, nurses received regular training informally and formally. However, there is no formal education to improve nurses’ NI competency. The rapid growth of e-Health has increased the demand for nurses with high NI competency in hospitals. Therefore, nurses require more continuing education to better meet requirements of their job.

### Sampling

The study population included all nurses (*N* = 330) working in the critical care settings mentioned above at the time of data collection. The sample size was calculated according to a pervious study and the sample size formula. Regarding α = 0.05, test power of 80%, and a large effect size (Cohen d = 0.7), the required sample size was 27 participants for each group (54 participants are needed for the two groups). Sixty nurses were recruited in the study using the stratified random sampling method (30 nurses in each group) with 10 % dropout probability. The studied samples were selected equally from each hospital. Stratified random sampling was appropriate to have a representative sample of the population under study. We first prepared three separate lists of nurses working in critical care settings of the three hospitals. Owing to the fact that the number of nurses in the three hospitals was almost equal, we selected 20 nurses from each hospital with the random number Table (10 nurses for the intervention group and 10 nurses for the control group). In total, 60 nurses were assigned into the intervention (*n* = 30) and control groups (*n* = 30). The inclusion criteria were nurses with a bachelor’s degree, at least 6 months of work experience in the critical care units as well as not being scheduled for a clinical shift on the days of the workshop. The exclusion criteria included being absent in one session and incomplete questionnaire. None of the nurses were excluded based on the inclusion criteria.

### Instruments

The instrument used in this study consisted of two questionnaires. The first one was about the nurses’ demographic and professional information, including gender, marital status, age, work experience, work experience in critical care settings, work position, shift work, history of attendance at research, information literacy skills, information-seeking skills, and computer skills training (Table 2).

The second was Nursing Informatics Competency Assessment Tool (NICAT) developed by Rahman [5] in the US in 2015 based on ANA standards (2008), TIGER recommendations (2009), and Benner’s *Dreyfus* model of skill acquisition (1984). NICAT can be utilized for evaluation of educational and training programs on NI competency. This tool has the potential to assess and evaluate all nurses during the new hire process, orientation, education, and clinical informatics system implementation. NICAT may not be applicable to bedside nurses with advanced degrees. The NICAT assesses NI competency with 30 items in three dimensions:

1. Computer literacy (10 items, items 1 to 10) shows the psychomotor skills to use computer tools, as well as knowledge of basic hardware and software functionality; these are all required for effective bedside nursing.
2. Informatics literacy (13 items, items 11 to 23) is the nurses’ abilities to recognize, retrieve, evaluate, and use information for patient care appropriately.
3. Information management skills (7 items, items 24 to 30) apply the data to support clinical decisions, documentation, data integrity, confidentiality, and security. Information management skill shows the value of information system in improving patient safety, quality, and outcome.

The NICAT is scored on a five-point Likert scale ranging from one to five: not competent (1 points), somewhat competent (2 points), competent (3 points), very competent (4 points), and expert (5 points). The overall score range of the instrument is 30–150. The higher the scores the higher the NI competency; a total score of 30 shows a novice nurse, a score between 31 and 59 represents advanced beginner, a score between 60 and 89 indicates a competent nurse, a score from 90 to 119 offers a proficient nurse, and a score between 120 and 150 is considered as an expert. Benner’s model outlines nursing skill acquisition from novice to expert. The novice learns the tools and thinks like a nurse. The advanced beginner applies clinical knowledge and judgments in delivering care. A competent nurse can use the tools and reason through issues familiar with the patient population. A proficient nurse can use all tools, recognize and anticipate patient needs. An expert nurse, in addition to all the above, has the vision to compose a team, look beyond the current moment, analyze system issues, and seek continuous improvements in practice [5].

Three groups, including the nursing informatics team, the department of education, practice, and research, and the hospital clinical outcome department validated the original NICAT. Clarity, readability, accuracy, question sequence, comprehensiveness of the questions, relevance, and appropriateness of the item were rated during the expert content validation and review. Abd El (2017) assessed the nursing informatics competencies of the critical care nurses. Researcher determined content validity with the help of information technology expert and confirmed the reliability using internal consistency. Cronbach’s alpha coefficient was 0.824 for each dimension and 0.846 for all items indicating sufficient internal consistency [13].

The authors of this study translated and validated NICAT in Iran. For cross-cultural adaptation, the original NICAT was accurately translated into Persian (forward translation). A proficient English translator did the backward translation of Persian version. Then, the translated version was matched with the original version. Nurses’ perceptions of the items were investigated to check the face validity of NICAT. Two medical informatics specialists and eight nursing faculty members confirmed the content validity of the adapted version of NICAT. Content validity was enhanced by using experts’ direct quotes, face-to-face discussions, and comments. Furthermore, 30 nurses participated in the pilot-test of the questionnaire and the Cronbach’s alpha coefficient was used to assess its reliability (α = 0.95).

### Data collection

The aim was to assess the effect of an educational program on the critical care nurses’ NI competency. Data were collected using an anonymous, self-reported, and structured questionnaire. To collect data, the first researcher referred to the study settings in different shift works, distributed the questionnaires among nurses of the intervention and control groups in the pretest (before workshop) and posttest stages (1 month after the workshop). She also trained participants how to fill out the questionnaires. To attain the highest response rate, the researcher spent appropriate time on data collection and coordination of workshop time with nurses in the intervention group. She established a friendly relationship with the participants and determined a deadline to deliver completed questionnaires. Moreover, she reminded the intervention group to attend the workshop in the scheduled time. It should be noted that all of the participants completed questionnaires during paid work time simultaneously with routine or traditional training programs in hospitals, except that the intervention group was provided with additional materials derived from workshop and the control group did not receive this educational program. In other words, the two study groups had equivalent conditions for work duties and attendance in other educational programs in hospitals. However, to increase internal validity of the study, researchers monitored the study conditions thoroughly to ensure that the intervention and control groups were identical in all aspects, except attending the educational program.

### Intervention procedure

A three-day workshop was held in 3 weeks (an eight-hour session on a day) in the informatics center located in Kerman University of Medical Sciences, with an adequate number of personal computers and internet access. The intervention group was divided into two groups of 15 people to increase the opportunity of engagement in the workshop. Therefore, all the workshops were held with the same structure and topics in 6 days.

To prepare and develop the workshop content, the researchers reviewed the literature to find necessary NI competencies for clinical nurses, and their educational needs in NI competency [4, 12–14, 30–32]. They discussed the extracted topics to achieve a consensus concerning goals, contents and teaching strategies. The literature revealed three key areas in NI competencies: computer literacy, informatics literacy, and information management skills applicable to the nursing care. These areas met ANA standards (2008) and TIGER recommendations (2009). The researchers employed the TIGER NI competencies as a guide and selected essential NI competencies. Nurses with a standard and consistent set of competencies can use HER better. The researchers also consulted with a multidisciplinary team, including three medical informatics specialists, two nursing faculty members, an expert in the field of medical education, three educational supervisors, and two critical care nurses. The team members presented their experiences and perspectives on required training and competencies of clinical nurses concerning nursing informatics in current practice, as well as teaching-learning activities and available educational programs for health informatics in study settings. In this step, the topics were developed using focus group discussion and then the team reviewed them for suitability, feasibility, applicability and relevance to the nursing workflow. Each item of the developed content was frequently reviewed and revised. After coming to an agreement on the NI competencies, the researcher drafted the content of workshop.

Two medical informatics specialists and six nursing faculty members who were not in the research group approved the content validity of the educational content. Few corrections were made based on their comments. Finally, a PhD in nursing and three experts in medical informatics taught the generated content as follows: lecture, demonstration, question and answer, slide presentation, hands-on exercise, group discussion and work, and online exercise (with examples of literature search using topics suggested by nurses), homework, video film, and educational CDs. The workshop in each day included training sessions in theory and practice. The topics presented in the workshop are shown in Table 1.

**Table 1:** Main topics presented in the workshop

Session 1 (in the first week)	Familiarity with and ability to use computer in the following areas: • Windows, data storing and processing, data access and security • Introducing different file formats, and key notes about advanced word processing functionalities, presenting slides and key notes (hyperlink, transition, slide master, design), managing reference • Using telecommunication, telenursing and software tools such as email, adobe connect, skype, and Lync • Managing computer systems security to protect data, devices, passwords and detect viruses • Demonstrating how to search for policies and procedures, just-in-time training materials • Demonstrating Internet tutorial and introducing databases and search engines, Google and key notes (translate- image- books- directory-scholar …), searching for policies and procedures and information, training just-in-time materials on the web
Session 2 (in the second week)	• Introducing and orientating variety of information sources including hard copies, electronic files • Demonstrating medical and nursing databases such as Current Nursing and Allied Health Literature)CINAHL(, PubMed, Scopus,… • Using search strategies in databases such as PubMed and Scopus • Searching articles in Persian databases such as Scientific Information Database)SID(, Medlib, Iranmedex, and Magiran • Demonstrating a variety of electronic search capabilities such as the ways to subscribe and receive free articles • Doing simple and advanced search, and conducting limited search based on the publication year, full text, keywords, Medical Subject Headings (MeSH), and using search operators such as AND, OR, NOT and etc. • Doing practical exercises. For example, retrieving related articles in databases such as PubMed and Scopus for “Intubate Patient Care” with related keywords and providing search results • Sending results of search through email to the professors for receiving feedback from them
Session 3 (in the third week)	• Presenting instances and practical exercises about the topics taught in the second session • Reviewing IT initiatives such as HIS and its various capabilities such as documenting care plan in the medical record, collecting and maintaining patient care data like laboratory data, the picture archiving and communication system, patient admission and discharge • Identifying human-computer interaction bottlenecks to enhance care quality, implementing HIT for higher care quality and patient-nurse communication. • Introducing the use of informatics tools for the design of effective care plan such as: nursing interventions classification (NIC), and nursing outcomes classification (NOC) • Correcting some flaws in the nursing care systems, data recovery and security, levels of safety and access to health information • Discussing about barriers to timely documentation, reviewing methods to improve documentation in an efficient and timely manner and providing egregious examples

### Statistical analysis

The data were analyzed in SPSS 21 using descriptive (frequency, percentage, mean and standard deviation) and inferential statistics (independent samples *t*-test, paired *t*-test, chi square test, and the analysis of covariance). The Kolmogorov-Smirnov test showed that the data followed a normal distribution. The significance level was considered ≤0.05.

## Results

### Demographic and professional information

All participants completed the educational program and returned the completed questionnaire (response rate = 100%). The results showed that most of the participants in the intervention and control groups were female (83.30%), married nurses (70.90, 73.30%) and aged 30–40 years (51.6, 35.5%). 35.70% of the participants in the intervention group had 11–15 years of work experience in critical care units; whereas, 36.60% of the participants in the control group had less than 5 years of work experience. Most of the participants in the control and intervention groups were not trained for research (96.80, 83.30%), information literacy (93.50, 83.30%), literature search and retrieval (96.80, 83.30%) as well as computer skills (87.10, 83.30%). Most of the participants tended to use databases moderately (54.80, 40.00%). Based on the chi-square test, no significant difference was found between the intervention and control groups in demographic and professional information (Table 2). Moreover, no significant difference was observed in the mean scores of NI competency between the study groups at the pretest stage. Independent samples *t*-test indicated homogeneity of the participants in the two study groups at the baseline (Table 3).

**Table 2:** Comparison of demographic and professional information of nurses in the intervention and control groups

Variables	Groups	Intervention		Control		*χ2*	*P*
		n	%	n	%		
Gender	male	5	16.70	5	16.70	0.003	0.99
	Female	25	83.30	25	83.30		
Age groups	< 30	10	32.3	11	35.5	2.11	0.347
	30–40	15	51.6	11	35.5		
	> 40	5	16.1	8	29		
Marital status	Single	9	29.10	8	26.70	1.06	0.58
	Married	21	70.90	22	73.30		
Work experience (year)	< 5	8	25.80	12	40	4.03	0.40
	5–10	4	12.90	4	13.30		
	11–15	12	41.90	6	20		
	16–20	4	12.90	4	13.30		
	> 21	2	6.50	4	13.30		
Work experience in critical care unit (year)	< 5	7	25	11	36.60	3.86	0.42
	5–10	9	28.60	8	26.60		
	11–15	11	35.70	9	28.60		
	16–20	2	7.10	1	3.60		
	> 21	1	3.60	1	3.60		
Work position	Head nurse	0	0	3	10	3.26	0.7
	Nurse	30	100	27	90		
Shift work	Fix	1	3.20	2	6.70	0.38	0.53
	Rotation	29	96.80	28	93.30		
Attendance at research training	Yes	1	3.20	5	16.70	3.10	0.07
	No	29	96.80	25	83.30		
Attendance at information literacy training	Yes	2	6.50	5	16.70	1.56	0.21
	no	28	93.50	25	83.30		
Attendance at literature search and retrieval training	yes	1	3.20	5	16.70	3.10	0.07
	no	29	96.80	25	83.30		
Attendance at computer skills training	yes	4	12.90	5	16.70	0.17	0.69
	no	26	87.10	25	83.30		
The tendency to use databases	low	2	6.50	8	26.70	4.62	0.09
	moderate	16	54.80	12	40		
	high	12	38.70	10	33.30

**Table 3:** Comparison of scores of NI competency and its dimensions between the intervention and control groups at pretest and posttest

Variables	Groups	Pre test *M* ± SD	Post test *M* ± SD	Mean difference	Size effect (Cohen d)	Statistic *t*^a^ & *p*
Informatics competency total	Intervention Control Statistic *t*^b^ & *p* Effect size (Cohen d)	78.79 ± 26.52 82.69 ± 19.29 *t* = − 0.45 *p* = 0.65 0.14	114.29 ± 20.68 81.76 ± 17.99 *t* = 6.54 ***p*** **= 0.001** 1.57	35.5 − 0**.**93	1.33 0.044	*t* = 7.71 ***p*** **= 0.001** *t* = − 1.39 *p* = 0.25
Computer literacy	Intervention Control Statistic *t*^b^ & *p* Effect size (Cohen d)	28.46 ± 10.28 28 ± 8.27 *t* = 0.194 *p* = 0.85 0.04	39.70 ± 6.78 28.03 ± 8.32 *t* = 6.05 ***p*** **= 0.001** 1.72	11.24 0.03	1.09 0.05	*t* = 7.06 ***p*** **= 0.001** *t* = 0.37 *p* = 0.71
Informatics literacy	Intervention Control Statistic *t*^b^ & *p* Effect size (Cohen d)	33.51 ± 11.45 34.70 ± 8.33 t = − 9.42 *p* = 0.67 0.10	48.77 ± 9.46 34.43 ± 7.79 *t* = 6.44 ***p*** **= 0.001** 1.51	15.26 − 0.27	1.33 0.03	*t* = 6.93 ***p*** **= 0.001** *t* = − 1.39 *P* = 0.30
Informatics management skills	Intervention Control Statistic *t*^b^ & *p* Effect size (Cohen d)	17.80 ± 6.78 19.88 ± 5.21 *t* = − 1.27 *p* = 0.27 0.30	25.80 ± 5.72 19.30 ± 5.40 *t* = 4.55 ***p*** **= 0.001** 1.13	8.00 − 0.58	1.17 0.11	*t* = 7.80 ***p*** **= 0.001** *t* = 1 *p* = 0.32

^a^ Paired *t*-test

^b^ Independent *t*-test

### Comparison of changes of NI competency in the intervention group

Table 3 shows that the total score of NI competency in the intervention group was at the “competent” level in pretest. The paired *t*-test revealed that the total score of NI competency in the intervention group increased statistically significantly and achieved the “proficient” level at the posttest with *t* = 7.71, *p* = 0.001, Cohen d = 1.33 (a very large effect size). All scores of NI competency dimensions increased statistically significantly in the posttest [computer literacy scores: *t* = 7.06, *p* = 0.001, Cohen d = 1.09 (a very large effect size), informatics literacy scores: *t* = 6.93, *p* = 0.001, Cohen d = 1.33 (a very large effect size), and informatics management skills scores: *t* = 7.80, *p* = 0.001, Cohen d = 1.17 (a very large effect size)]. These significant differences show that the training program improved the total scores of NI competency and its dimensions in the intervention group from the “competent” to “proficient” levels with a very large effect size. The highest mean difference was associated with the informatics literacy dimension (mean difference = 15.26), while the lowest mean difference was associated with the informatics management skills dimension (mean difference = 8.00). The mean differences show that training program had the highest impact on the informatics literacy dimension and the lowest impact on the informatics management skills.

### Comparison of changes of NI competency in the control group

A comparison of the pretest and posttest scores in the control group revealed no significant improvement in NI competency and its dimensions; the scores were at the “competent” level in both stages (*t* = − 1.39, *p* = 0.25).

### Comparison of changes of NI competency between the intervention and control groups

Table 3 shows the level of NI competency and its dimensions in both groups before and after the intervention. In the pretest phase, independent sample t-test showed no statistically significant difference in the scores of NI competency between the intervention (79.78 ± 26.52) and control (82.69 ± 19.29) groups (*t* = 0.45, *P* = 0.65). Moreover, the comparison of the intervention and the control groups revealed that the scores of NI competency and its demotions were at the “competent” level for both intervention and control groups. In the posttest phase, a statistically significant difference was observed between the intervention (114.29 ± 20.68) and control (81.76 ± 17.99) groups in total score of the NI competency with *t* = 6.54, *p* = 0.001, Cohen d = 1.57 (with a very large effect size). Data analyses also revealed that all scores of the NI competency dimensions in the intervention group improved statistically significantly in the posttest compared with the control group [computer literacy scores: *t* = 6.05, *p* = 0.001, Cohen d = 1.72 (a very large effect size), informatics literacy scores: *t* = 6.44, *p* = 0.001, Cohen d = 1.51 (a very large effect size), informatics management skills score: *t* = 4.55, *p* = 0.001, Cohen d = 1.13 (a very large effect size)].

In Table 4, we used covariance analysis test to control the impact of pretest on the NI competency of nurses. Concerning the pretest effect, the results showed a statistically significant difference between the control and intervention groups in the total posttest scores of NI competency and its dimensions. These results are also consistent with the results of Table 3.

**Table 4:** Summary of covariance analysis for the two groups of control and intervention

Variables		df	Mean square	F	*p*-value
Informatics competency total	Corrected model	2	10,618.1	42.71	**< 0.001**
	Intercept	1	10,459.56	42.07	**< 0.001**
	Pre test	1	8607.59	34.62	**< 0.001**
	Group	1	13,915.42	55.97	**< 0.001**
	Error	57	248.6		
Computer literacy	Corrected model	2	1982.71	77.17	**< 0.001**
	Intercept	1	1553.39	60.49	**< 0.001**
	Pre test	1	1923.77	74.82	**< 0.001**
	Group	1	1940.92	75.49	**< 0.001**
	Error	57	25.71		
Informatics literacy	Corrected model	2	2108.58	39.26	**< 0.001**
	Intercept	1	2205.53	41.07	**< 0.001**
	Pre test	1	1528.43	28.46	**< 0.001**
	Group	1	2924.8	54.47	**< 0.001**
	Error	57	53.69		
Informatics management skills	Corrected model	2	702.8	50.56	**< 0.001**
	Intercept	1	557.5	40.11	**< 0.001**
	Pre test	1	916.14	65.91	**< 0.001**
	Group	1	725.72	52.21	**< 0.001**
	Error	57	13.89		

## Discussion

### Principal results

This study evaluated the effect of a training program on NI competency of the critical care nurses in Iran. The results indicated that the program significantly improved NI competency and its dimensions in the intervention group compared with the control group, with a large effect size. The NI competency of the intervention group promoted from “competent” level in pretest to “proficient” level after the training program. Several studies in different countries reported that nurses’ NI competency was at the competent level before attending any educational intervention [13, 33–36]. However, other studies indicated that nurses’ NI competency was low [14, 37, 38]. Some other studies reported the nurses’ NI competency at the expert and proficient levels [2, 39, 40].

Several studies in different countries evaluated impact of the training programs on NI competency of the participants and reported its improvement [4, 27, 30, 41]. Pereira et al. in Spain used three technological tools (power point, open meetings and babelium) to improve NI competency of the nursing students. Researchers also used self-, peer-, and teacher assessments to assess the program effectiveness. They reported that NI competency of the students improved at the end of program, and more than 53% of the students reported the development of their creativity. There was an acceptable correspondence between self- and peer-assessments [42]. In another study, researchers designed a blended course to develop nursing students’ informatics competency. This project successfully improved nursing students’ informatics competency. The majority of students perceived the teaching strategy of blended-learning positively that was used throughout the course [31]. Rajalahti et al. in Finland found that NI competency improved in nurse educators and nurses who completed an educational project. In post-project, nurses were better prepared and confident to use the electronic health record (EHR), make better clinical decisions and provide better patient care. The nurse educators also better mastered EBP and used nursing process models in their work [28]. Iranian studies also reported a significant increase in NI competency of the faculty members [43] and nurses in ICUs after attending the educational program compared with the control group [17]. Similar results may be due to application of a similar training program and advancement of HIT in clinical setting and everyday life, which has prompted individuals and nurses to acquire and enhance their NI competency. Moreover, current nurses are novices in technology and are known as digital immigrants. Therefore, they have to learn and adopt many aspects of the new technology. If digital immigrants really want to reach digital natives, they will have to change. Therefore, nurses should be equipped with NI competencies in formal and informal training inside and outside the organization. On the other hand, managers and policymakers in the healthcare system have emphasized this issue and strived to provide the necessary infrastructure to empower the staff nursing.

However, a study examined the effect of training on NI competency, which does not support our study. The researchers reported no significant difference between the intervention and control groups in nurses’ NI competency after the intervention. As they reported, both groups improved their information seeking skills in the posttest [44].

The results indicated that the highest mean difference in the intervention group was associated with the informatics literacy dimension and the lowest mean difference was associated with the informatics management skills dimension. A study also showed that at the post-test stage, the level of nurses’ NI competency promoted in the intervention group, and educational intervention had the most impact on the dimension of informatics literacy [28]. Conversely, in another study which assessed the effect of a project on NI competency nurses, the lowest mean difference was related to the informatics literacy, while the highest mean difference was attributed to information management [4].

Our results showed that NI competency and all its dimensions remained at the “competent” level in the control group and no significant difference was observed between the pretest and posttest scores. It can be said that nurses’ NI competency is a determinant factor in successful application of clinical information systems. The current conditions and the hospitals emphasis on application of HIT in different clinical settings required the nurses to seek NI competency. Karimi et al. [45] as well as Raei and Haseli [43] showed that the scores of NI competency in the control group did not have a significant difference before and after the educational course. Esfandani et al. investigated critical care nurses and reported that the scores of information seeking skills were low in the control group with no significant difference in their scores before and after the intervention [17]. Conversely, a study also reported that the posttest score of information skills increased in the control group [44].

The discrepancy between results of the above-mentioned studies and the present study can be due the differences in community, sampling, randomization and matching of the groups, educational backgrounds, clinical and experiential learning environment, participants’ previous skills, nursing education preparation levels, study design and educational content, data collection tools and conditions. For example, recommendations of the TIGER Initiative were fundamental for the development of data collection tool and educational content of our project.

### Limitations

This study had some limitations that need to be addressed. First, this study included a limited training time; the training time should be increased for more topics and coverage of NI competency during the project. Multiple class offerings will allow more nurses to enroll in such a project and thereby increase the generalizability and power of the results. Second, we used self-report tool to assess NI competency; therefore, the evaluation has been conducted at a low level of Kirkpatrick’s model such as reaction, learning. Future research may look at the effectiveness of training program at higher levels such as in behaviors, results and outcomes [46, 47]. We suggest the evaluation of educational interventions related to NI competency at all levels of Kirkpatrick’s model to provide the evidence required to be aware of future initiatives. Third, evaluation of NI competency might have been influenced by the social desirability bias inherent in the self-assessment and self-report methodology applied in this study, because participants tended to overrate their levels of NI competency; therefore, the data might not reflect the actual level of nurses’ NI competency. Future studies can be performed using blended methods of competency evaluation such as of Kirkpatrick’s four-level model and 360-degree method to help determine the actual NI competency. Evaluation of NI competency is strengthened through a combination of different approaches, which are suitable for evaluating competency. The Kirkpatrick model has contributed to the model popularity and perseverance as the dominant model of competency evaluation. In addition, the comparative results of the 360-degree evaluation can help nurses better understand their competencies, and they are more motivated to advance professionally in the organization. Moreover, this evaluation will help the nurse manger better understand the training and development needs of nurses and plan for them. Finally, data collection was conducted 1 month after the intervention. Follow-ups with 3–6 month intervals are recommended to have more accurate results, compare the results to determine the long-term impact of training, and to assess the effect of educational courses on NI competency.

## Conclusions

This project could significantly improve critical care nurses’ NI competency and its dimensions. Recently, the need to continue NI competency development has been recognized by national and international healthcare system and the nursing community. It highlights the importance of initiative approaches to promote NI competency of the nurses in all fields. The results suggest that nurse managers, decision-makers, nurse educators, and authorities of clinical settings should organize appropriate interventions and training programs with the help of informatics specialists to improve nurses’ NI competency particularly in the field of information management skills. These strategies may include assessment of training needs, design and modification of curriculum for integrating topics like NI competency. The training process used in this project can also be utilized as an example for designing training programs for nurses and other health professionals. Persian version of the NICAT that was validated and translated in this study can be beneficial in NI competency assessment and examination of its status and gaps of nurses’ competency in Iranian healthcare. In addition, nurse managers should highlight the importance of well-competent and efficient nurses in providing patient-centered evidence-based care. They should provide required resources to promote NI competency of nurses. It is essential to strengthen the collaboration and interaction between the universities and hospitals because nursing schools are in the best position to develop NI competency in future nurses. With integrating NI competency into nursing education and using blended courses to develop nursing students’ NI competency, future nurses will be able to effectively balance HIT with clinical demands. Further detailed research is also recommended to explore NI competency in healthcare and evaluate effectiveness of different approaches and models to enhance the NI competency of nurses in Iran and other cultures and contexts.

## Data Availability

The data are available upon request to the corresponding author after signing appropriate documents in line with ethical application and the decision of the Ethics Committee.

## Abbreviations

ANA: American Nurses Association
CCU: Coronary care unit
CINAHL: Cumulative Index to Nursing & Allied Health Literature
EBP: Evidence-based practice
EHR: Electronic health record
ICU: Intensive care unit
IT: Information technology
HIT: Health information technology
HIS: Health Information System
NI: Nursing informatics
NICAT: Nursing Informatics Competency Assessment Tool
NIC: Nursing interventions classification
NOC: Nursing outcomes classification
MeSH: Medical Subject Headings
TIGER: Technology Information Guiding Education Reform
SID: Scientific Information Databases
